# Scale‐Up Synthesis of Fragrant Nitrogen‐Doped Carbon Dots from Bee Pollens for Bioimaging and Catalysis

**DOI:** 10.1002/advs.201500002

**Published:** 2015-02-26

**Authors:** Jia Zhang, Yue Yuan, Gaolin Liang, Shu‐Hong Yu

**Affiliations:** ^1^ Division of Nanomaterials and ChemistryHefei National Laboratory for Physical Sciences at MicroscaleDepartment of ChemistryCollaborative Innovation Center of Suzhou Nano Science and TechnologyUniversity of Science and Technology of ChinaHefeiAnhui230026P.R. China

**Keywords:** bee pollens, carbon dots, catalysis, fluorescence, hydrothermal synthesis

## Abstract

**Fragrant nitrogen‐doped carbon dots of gram scale** can be prepared from commercial bee pollens by a hydrothermal process. These carbon dots of 1–2 nm in size show promising applications in cellular imaging and catalysis/photocatalysis.

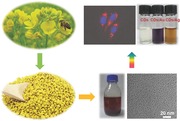

Since its serendipitous discovery,^1^, ^2^ photoluminescent carbon nanoparticles (or carbon dots, CDs) have aroused intense interests and soon become a star material among a diversity of nanomaterials.^3^ One of the most fascinating properties of CDs is their multicolor luminescence in the visible region. Compared to the widely used cadmium‐based semiconductor quantum dots, the CDs are much less toxic and exhibit tunable emissions upon varied excitation. Classified by the starting carbon sources, there are three dominant approaches for the preparation of emissive CDs, including acidizing carbon materials,^4^ carbonizing natural bioresources,^5^ and thermal condensation then carbonization of small organic molecules.^6^ The main drawback of the first approach lies in its complicated procedures including the usage of strong acid, dialysis, and surface passivation. The other two approaches are essentially alike, both feasible for one‐pot synthesis without additional surface functionalization, while the number of biomass is much more diverse in species than that of small molecules utilized. To date, a variety of green resources have been adopted to generate CDs for applications in sensing[[qv: 5a]],^7^ and cellular imaging;[[qv: 5b,d,g]],^8^ however, the mass conversion ratio was rarely concerned (Table S1, Supporting Information). Besides, none attempt of scale‐up synthesis has ever been performed. It should be noted that the strategy of macroscopic preparation is of vital importance for the future widespread utilities of CDs.

Herein, we report a green, versatile, and scalable route to prepare CDs in large scale from commercial bee pollens with high production yield and extend their applications for multicolor cellular imaging and homogeneous catalysis. We initially collected some natural pollen from a bunch of lilies to test the possibility of generating CDs. After hydrothermal treatment at 180 °C for several hours, we obtained some fragrant brown suspension after filtration of the product to remove the insoluble residual. It emitted much brighter blue‐green color than the original solution under excitation at 365 nm, suggesting the formation of CDs. In view of the repeatability and scalability of the synthesis, we substituted natural pollen with commercial bee pollens.

Bee pollen is a mass of pollen packed by worker honeybees into granules used for feeding its larvae in the early stage of development and producing royal jelly. For centuries bee pollens have been favorably consumed as diet supplementation since they are rich in biomolecules, including sugars, proteins, amino acids, and nucleic acids.^9^ There are several noteworthy merits in producing CDs from bee pollens. First, the variety of bee pollens is abundant in markets and the price can be affordable. For the bee pollens used in this study, each bottle of bee pollen weighted 500 g costs 35 Chinese yuan on average, thus every 1 g costing only 0.07 Chinese yuan, or 0.011 dollar. Second, the synthesis is green, remarkably simple, highly reproducible, and capable to be scaled up to large volumes. Third, the production yield is fairly high. At least 3 g of CDs can be prepared from 10 g input of the raw material, manifesting a conversion ratio of ≈30% (w/w). Fourth, the CDs are fragrant and doped with a relatively high content of nitrogen, exhibiting fluorescence quantum yields (QYs) between 6.1% and 12.8%, depending on the species of bee pollen and reaction time. Fifth, the CDs display excellent biocompatibility. Last, with their sizes being 1–2 nm, the dots manifest superior catalysis and photocatalysis towards the reduction of noble metal ions in aqueous solution.

Three kinds of bee pollens were utilized in our study, namely rapeseed flower bee pollen, camellia bee pollen, and lotus bee pollen (Figure S1, Supporting Information). To be simplified, CDs derived from the three bee pollens are individually denoted as r‐CDs, c‐CDs, and l‐CDs, with respect to rapeseed flower, camellia, and lotus, respectively. **Figure** **1**a shows the optical absorption spectrum of diluted solution of r‐CDs after hydrothermal treatment at 180 °C for 24 h. The elemental analysis (Table S2, Supporting Information) shows that the content percentage of carbon in the final product increases while that of oxygen minimizes relative to those in the bee pollen, providing definitive evidence for the carbonization of the molecule precursors within. This is reflected by a well‐defined absorption peak centered at 280 nm consistent with the representative π–π* transition of aromatic carbon. Moreover, there is an appreciable increase of nitrogen content in the CDs, suggesting the doping of nitrogen in the carbon structure during condensation reactions. In vivid contrast, the yellow turbid suspension of bee pollen changes to brown transparent solution of CDs after purification, as shown in Figure 1c. Like many other CDs,[[qv: 2a]],[[qv: 4a]],[[qv: 5e]],^10^ the fluorescence emission of the r‐CDs is excitation wavelength dependent, tunable from 425 to 505 nm upon excitation from 340 to 450 nm, enabling the observation of visible color shift from blue through cyan to green (Figure 1 b,d). The fluorescence QY upon excitation at 380 nm was calculated to be 9.1%, using quinine sulfate as the reference. Syntheses of CDs utilizing the other two bee pollens were also successful, resulting in similar absorption profiles and fluorescent spectra (Figure S2, Supporting Information). The QYs of c‐CDs and l‐CDs were 8.9% and 6.1%, respectively. The appreciable decrease of quantum efficiency for l‐CDs may be mechanistically related to its lower doping of nitrogen (4.28%) compared with those of r‐CDs (7.10%) and c‐CDs (6.67%) (Table S2, Supporting Information). Unlike the controversy in the emission mechanism of graphene quantum dots, it is now increasingly adopted that radiative recombinations of the surface‐confined electrons and holes are responsible for the fluorescence of CDs.^11^ Electrons trapped by the newly formed surface states arising from nitrogen doping will facilitate a high yield of radiative recombination, resulting in higher fluorescence QY.[[qv: 6g]] More than that, there are researches finding that a greater nitrogen content in CDs leading to larger emission,^12^ which is in agreement with the present study.

**Figure 1:**
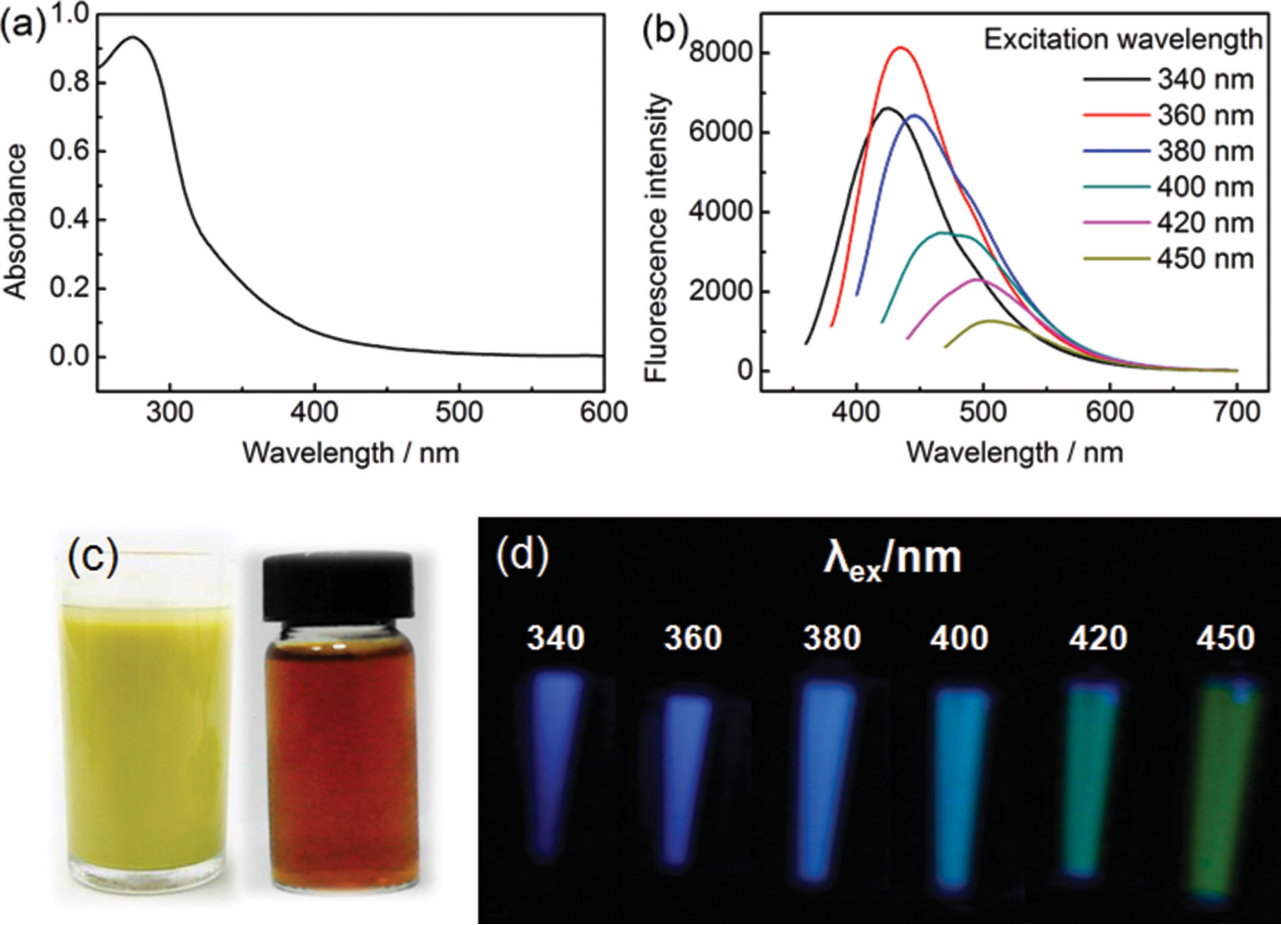
a) Absorption spectrum and b) wavelength‐dependent fluorescent emission spectra of diluted suspension of r‐CDs. The reaction volume was 40 mL and the feeding mass of bee pollen was 1.0 g. c) Digital photographs of suspension of rapeseed bee pollen (left) and as‐prepared solution of r‐CDs (right). d) A colorful demonstration of emission of r‐CDs solution under specific excitation wavelength.

The reaction time is vitally important for the dehydration and carbonization of the ingredients of bee pollens. Take the synthesis of r‐CDs as an example. We prepared r‐CDs in shorter reaction times (3, 6, and 12 h) and found that the fluorescence intensity increased linearly with time but the emission wavelength remained the same, when excited at 360 nm (Figure S3a,b, Supporting Information). The QYs for the less carbonized dots were calculated to be 5.0%, 5.2%, and 6.1%, respectively. This suggests that the CDs with higher quantum efficiency might be produced by prolonging the reaction beyond 24 h. Then we extended the reaction up to 36 and 48 h and recorded the fluorescent spectra of the samples (Figure S3c,d, Supporting Information). It showed that the fluorescence intensity was still in proportion to the reaction time, but with a much lower slope of variation. The QYs of r‐CDs prepared for 36 and 48 h were 10.0% and 12.8%, and the production yields were both 38%, a little higher than that of dots prepared for 24 h (31%). To obtain a higher QY within a shorter time, we selected 24 h as an appropriate time for the hydrothermal reaction. **Figure** **2** shows transmission electron microscope (TEM) images of CDs derived from the three bee pollens. All of the CDs were well distributed without aggregation and had mean particle diameters of 1.7, 1.2, and 1.1 nm for r‐CDs, c‐CDs, and l‐CDs (Figure S4, Supporting Information), supposing they are uniformly spherical. A representative atomic force microscopy (AFM) image of the r‐CDs showed densely packed dots on the mica substrate with particle heights from 1.8 to 2.3 nm (Figure S5, Supporting Information), which met exactly with the size range by TEM measurement. The equivalence of particle height with size differentiates the CDs from their counterpart, the graphene quantum dots, which usually have larger size over height.^13^ In comparison with the CDs in literature fabricated from other biomass (Table S1, Supporting Information), the CDs from bee pollens are the smallest in size. A high‐resolution TEM (HRTEM) image of the r‐CDs (Inset of Figure 2b) clearly exhibits lattice fringes with interplanar spacing of 0.18 nm, corresponding to the [102] facet of graphitic carbon.[[qv: 5b]],^14^ In the meantime, the powder X‐ray diffraction (XRD) patterns of the three CDs similarly showed a broad peak at 20° (Figure S6, Supporting Information). The XRD results, along with HRTEM data, infer that the CDs possess crystalline cores and amorphous surfaces.^15^ Alike the CDs from soy milk,[[qv: 5f]] the Raman spectrum of the CDs failed to be obtained when excited at wavelengths from visible to near‐infrared region, probably due to strong fluorescence background. The CDs were remarkably stable in suspension without little precipitation, even though their net charges were near zero (0.54, −0.46, and −1.07 mV with respect to r‐CDs, c‐CDs, and l‐CDs), according to the zeta potential measurements. Fourier transform IR (FTIR) spectrum verified the presence of amino group and carboxyl group embedded in the three CDs (Figure S7, Supporting Information), a manifestation of their zwitterionic characteristics, so the nearly neutral charge state implies that the side of positively charged amino group counterbalances the side of negatively charged carboxyl group. Besides, we observed no obvious difference in FTIR spectra among the three CDs, indicating that the chemical structures of the CDs are similar. The heteroatom nitrogen was further identified by X‐ray photoelectron spectroscopy technique (Figure S8, Supporting Information). A deeper survey of binding energy reveals two types of nitrogen, i.e., C−N−C (399.5–399.7 eV) and N−H (401–401.4 eV).[[qv: 5g]],^16^ This is consistent with the FTIR result. In addition, three types of carbon (C−C, C−O/C−N, and C=O) and two types of oxygen (C=O and C−O) are demonstrated after deconvoluting the individual spectrum for all CDs, which are comparable with those in literature.[[qv: 5g]],^16^, ^17^

**Figure 2:**
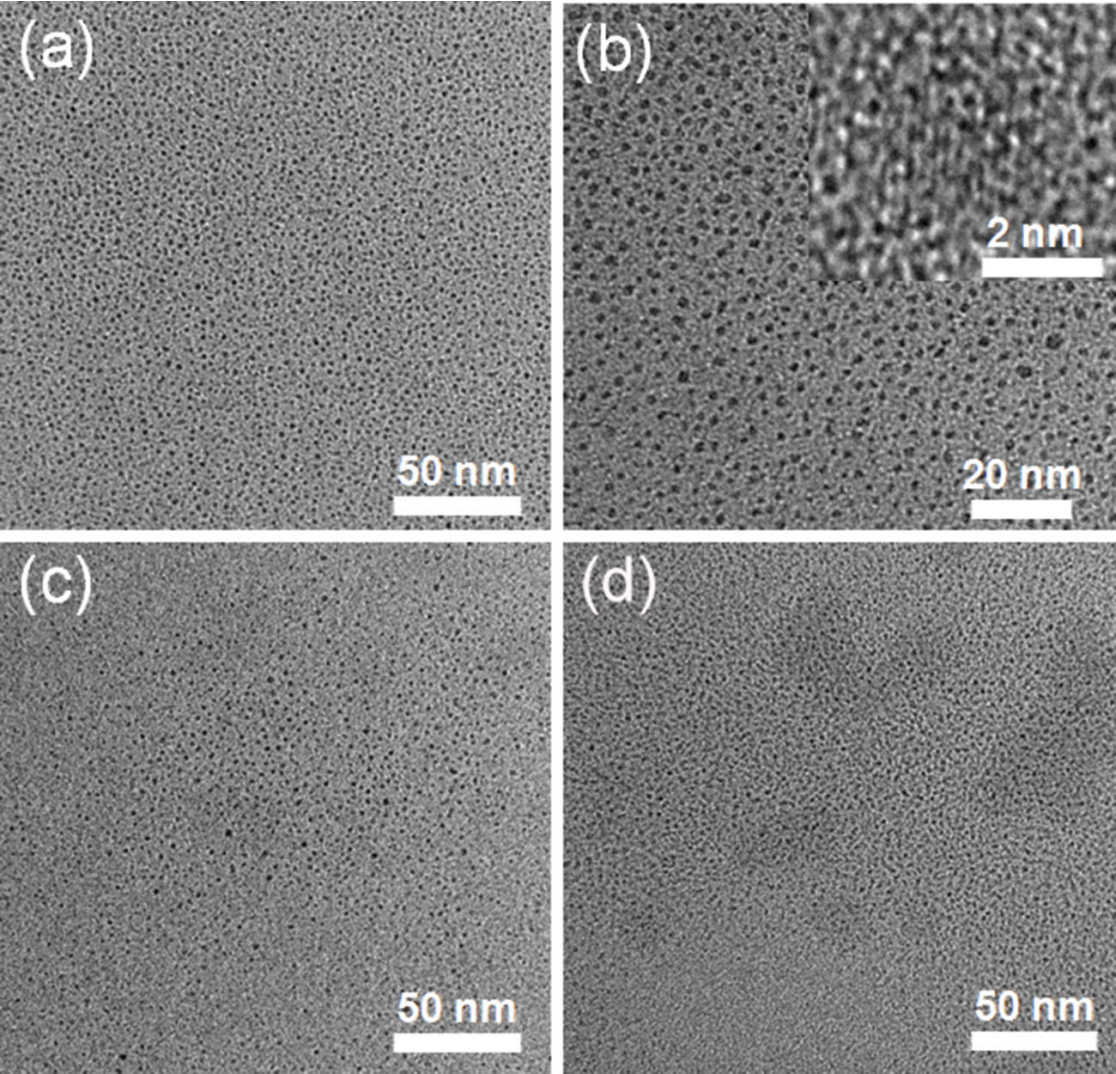
TEM and high‐resolution TEM images of a,b) r‐CDs, c) c‐CDs, and d) l‐CDs. The reaction volumes were 40 mL, the feeding mass of bee pollens were 1.0 g, and the reaction times were all 24 h.

Large‐scale synthesis of CDs is highly desirable for their prospective applications in bioimaging,^18^ biomedicine,[[qv: 8b]] sensing,^7^ photocatalysis,^19^ and energy conversion.^20^ Until now, there are few methods reported for macroscopic synthesis,^21^ and while the methods relying on hydrothermal treatment are capable of performing macroscopic synthesis, neither of them has ever attempted to do it. Moreover, the majority of the current hydrothermal methods using biomass as carbon resources do not mention the production yield. Using rapeseed bee pollen as the precursor, we prepared four CDs by gradually increasing the reaction volume, i.e., 20, 40, 80, and 400 mL, with feeding mass increasing in proportion (0.5, 1, 2, and 10 g). It is better to denote these four CDs as r‐CDs‐20, r‐CDs‐40, r‐CDs‐80, and r‐CDs‐400, respectively. **Figure** **3**a is a digital photograph of the four suspensions of CDs, showing no discernible color difference among them. After separating the indispersible matter by natural sedimentation and vacuum filtration, we extracted 10 mL of solution from each CDs suspension, followed by freeze drying, and obtained 73, 78, 80, and 71 mg of brown powder with respect to feeding mass of 0.5, 1, 2, and 10 g, respectively. Therefore, the production yield weights 30.2% ± 1.7%, which is much higher than those based on coffee grounds and egg (Table S1, Supporting Information). Moreover, the mean production yield with respect to synthesis of r‐CDs‐40 for five times weights 30.8% with standard deviation of 1.1%. Although more repetitive experiments entail to be performed, such small standard deviations in production yield among single batches for same reaction volume as well as for different reaction volumes may suggest that this approach is highly reproducible. It should be noted that the preparation of c‐CDs and l‐CDs also has excellent reproducibility. TEM studies show that the particle sizes of r‐CDs‐20, r‐CDs‐80, and r‐CDs‐400 are comparable with that of r‐CDs‐40 (Figure S9, Supporting Information), indicating that enlarging reaction volume has no meaningful influence on the size of CDs. The absorption spectra exhibited similarly well‐defined peaks at around 280 nm, irrespective of the reaction volume, and meanwhile, the fluorescence emission at 435 nm with excitation at 360 nm were observed for all CDs (Figure 3b). To be more specified, the emissions of the other three CDs were also dependent upon the excitation wavelength, reaching the highest at 360 nm excitation (Figure S10, Supporting Information). The QYs for the r‐CDs‐20, r‐CDs‐80, and r‐CDs‐400 were 9.5%, 10.5%, and 8.8%, respectively, with an average of 9.5% when including 9.1% for the r‐CDs‐40, which displays comparable with these of CDs from silk,^14^ banana juice,^22^ egg,[[qv: 5e]] and pomelo peel,[[qv: 5a]] but better than those of CDs from coffee grounds^23^ and soy milk[[qv: 5f]] (Figure S1, Supporting Information). On the basis of the above results, it is fair to say that hydrothermal treatment of bee pollens at relatively low temperature provides an environmentally benign and highly reproducible way to prepare nitrogen‐doped CDs with excellent fluorescence properties in a macroscopic manner.

**Figure 3:**
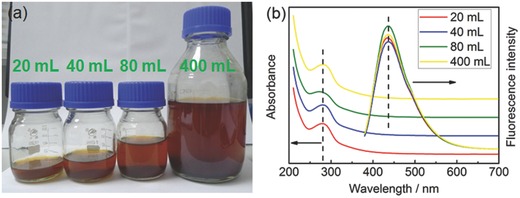
a) A photograph of suspensions of r‐CDs synthesized at increasing volumes for 24 h. b) Absorption spectra and fluorescent emission spectra on 360 nm excitation for suspensions of r‐CDs synthesized at specific volumes.

To get insight to the physicochemical properties of the CDs, we studied the fluorescence lifetime first by time‐resolved fluorescent measurement. Irrespective of the kind of bee pollen, the fluorescence lifetimes of all three CDs took on biexponential decay, with average individually being 7.28, 7.54, and 7.61 ns for r‐CDs, c‐CDs, and l‐CDs (Table S3, Supporting Information). The lifetimes are almost the same for the three CDs, suggesting that their energy level structures of the fluorophore are alike. This is consistent with the FTIR results. From the concentration‐dependent absorption spectra, we can obtain the mass absorptivity of the CDs, which is 11.52 L g^−1^ cm^−1^ (Figure S11a,c, Supporting Information). On the other hand, the emission intensity of the CDs increased but not linearly with increase of concentration and it dropped down to zero at high concentration, i.e., 7.5 mg mL^−1^, because of reabsorption leading to self‐quenching (Figure S11b,d, Supporting Information). The CDs showed higher fluorescence intensity in all tested hydrophilic solvents than in water (Figure S12, Supporting Information), while they could not be dispersed in hydrophobic solvents, such as chloroform and hexane. At both ambient temperature and 4 °C, the suspensions of CDs in dilution were stable without any noticeable aggregation for several months. The fluorescence intensity of the CDs was not sensitive to change in temperature or solution pH (Figure S13a,b, Supporting Information), and more interestingly, the CDs were immune to many metal ions (Figure S14a, Supporting Information), including Cu^2+^ and Hg^2+^, which are commonly the fluorescent quencher for CDs.^24^ Likewise, a variety of inorganic anions caused no meaningful influence on the fluorescence of the CDs (Figure S14b, Supporting Information), but there were something which could vary the fluorescence, such as Cr_2_O_7_^2−^, MnO_4_^−^, OCl^−^/HOCl, and NaBH_4_ (Figure S14c, Supporting Information). For the first two anions, the fluorescence decrease is known to be attributed to the inner filter effect,^25^ and for the last two, the changes are assumed due to variations in the surface states of CDs after oxidation by OCl^−^ 26 or reduction by NaBH_4_.^27^ Same as other CDs, the CDs possessed outstanding stability against continual photoirradiation (Figure S13c, Supporting Information), and moreover, the CDs were stable in 1 mol L^−1^ NaCl solution as in water (Figure S13d, Supporting Information). In view of the ultrasmall size, the immunity to the environmentally ordinary ions, and the stable fluorescent properties, we may envision a good opportunity for the CDs to be used in bioimaging. Before this, the cytotoxicity of the CDs was evaluated in vitro. The proliferation tests showed that the CDs were safe to cells when their concentration reached 0.5 mg mL^−1^ (Figure S15, Supporting Information), manifesting excellent biocompatibility of the CDs. **Figure** **4** shows the fluorescence microscope images of LoVo human colon carcinoma cells stained with r‐CDs (0.5 mg mL^−1^). Multicolor fluorescence were clearly observed under different excitation wavelengths from the cytoplasm of cells around the nuclei (also Figure S16, Supporting Information), which should be ascribed to the entering of CDs probably by endocytosis. The fluorescence could last for minutes upon constant laser irradiation, as another proof of photostability of the CDs.

**Figure 4:**
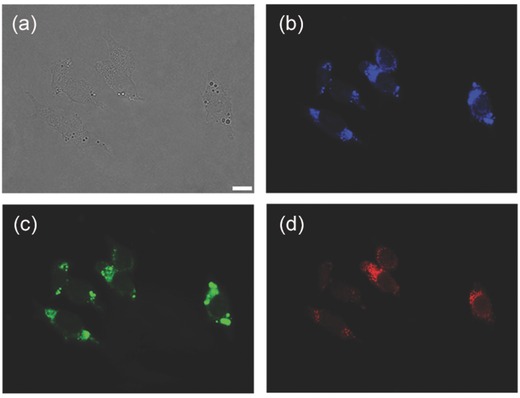
a) Bright‐field image and b–d) fluorescent images of LoVo cell after incubation with r‐CDs (0.5 mg mL^−1^) for 12 h at 37 °C. Fluorescent images were captured under b) UV, c) blue, and d) green excitation. Scale bar: 10 μm.

In addition to cellular imaging, we further explored its catalytic properties of the CDs by probing into the reduction of chloroaurate and silver ions at room temperature. The growth of gold nanocrystals was accomplished in the additional presence of sodium citrate, and the formation of silver nanocrystals was performed under sunshine irradiation. After reaction, the suspension of CDs in faint yellow turned to solution of CDs/Au in deep purple or CDs/Ag in brown yellow (**Figure** **5**a). In contrast to that of CDs, the optical spectra clearly exhibited increase of absorbance after reaction for both solutions (Figure 5b), indicating the generation of nanoparticles. The plasmonic peak characteristic of gold nanocrystals was centered at 535 nm for CDs/Au, while on the other hand, the spectrum of CDs/Ag showed broad light absorption without obvious plasmonic peak, which is assumed due to electric field enhancement at the gap between the silver nanocrystals.^28^ The catalytic role of CDs was identified by the control sets of sunshine‐irradiated AgNO_3_ solution or HAuCl_4_ solution in the sole presence of CDs or sodium citrate (Figure 5c,d). For both reactions, the emission intensity of CDs solution was greatly decreased (Figure S17, Supporting Information). Based on this finding, a turn‐on fluorescent sensor for cyanide ion is under investigation. TEM images of the CDs/Au and CDs/Ag showed that most of Au and Ag nanocrystals were larger than 20 nm in size with irregular morphology and both manifested distinguishing lattice fringes of 0.23 nm (Figure S18a–d, Supporting Information). The powder XRD patterns (Figure S18e,f, Supporting Information) revealed the face‐centered cubic structure of Au and Ag nanocrystals. Of note is the different status for the presence of CDs. CDs existed freely from contact with Au nanocrystals for CDs/Au but connected in a matrix to support Ag nanocrystals for CDs/Ag. Such difference in the particulate state for CDs/Au and CDs/Ag implies different applications such as in sensing,^29^ surface enhanced Raman scattering,^30^ catalysis,^31^ or photocatalysis.^32^

**Figure 5:**
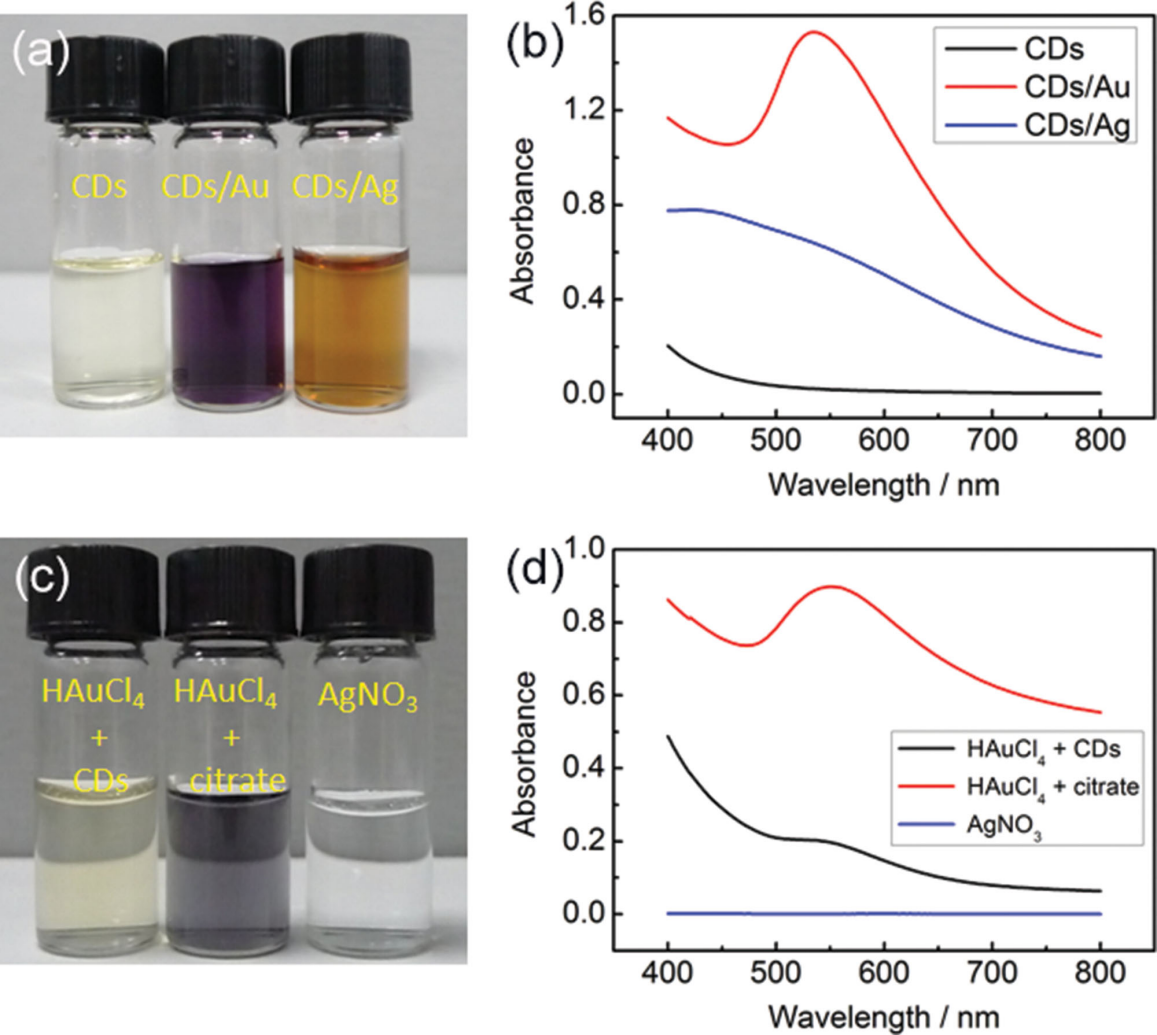
a) A photograph showing suspensions of r‐CDs, r‐CDs/Au, and r‐CDs/Ag and b) the corresponding absorption spectra. c,d) A photograph of solutions and the corresponding absorption spectra for the control sets.

In summary, we have demonstrated a scale‐up hydrothermal route to prepare nitrogen‐doped CDs of ultrasmall size by using commercial bee pollens as carbon resources. It is ecologically green, and because of its high reproducibility, the synthesis can be facilely scaled up to large volumes without affecting the mass conversion ratio appreciably. Owing to the abundant biomolecules within bee pollens, at least 3 g of CDs can be produced from 10 g of starting material, manifesting a high production yield. In vitro cytotoxicity tests show that the CDs are safe to cells when their concentration reaches 0.5 mg mL^−1^, and the relatively high QY of the CDs realizes the illumination of multicolor fluorescence on an inverted fluorescence microscope. In addition, the reduction of metal ions catalyzed by the CDs in homogeneous solutions has been performed, generating CDs‐metal nanocrystals with different particulate state, depending on the exact way of catalysis used. Using commercial bee pollens provides an economical and eco‐friendly approach to prepare photoluminescent CDs in a macroscopic and highly reproducible manner, which we believe to be remarkably significant for their promising widespread applications in bioimaging and light‐emitting devices and so on.

## Supporting information

As a service to our authors and readers, this journal provides supporting information supplied by the authors. Such materials are peer reviewed and may be re‐organized for online delivery, but are not copy‐edited or typeset. Technical support issues arising from supporting information (other than missing files) should be addressed to the authors.

Supplementary
