# Supplementary material for: Scale‐Up Synthesis of Fragrant Nitrogen‐Doped Carbon Dots from Bee Pollens for Bioimaging and Catalysis
Source: Adv Sci (Weinh). 2015 Feb 26;2(4):1500002. doi: 10.1002/advs.201500002 (PMC5115353; doi:10.1002/advs.201500002)
Supplement: Supplementary file 1 — Supplementary [file ADVS-2-0h-s001.pdf]

Copyright WILEY-VCH Verlag GmbH & Co. KGaA, 69469 Weinheim, Germany, 2015.

ADVANCED SCIENCE
Open Access

Supporting Information

for *Adv. Sci.*, DOI: 10.1002/advs.201500002

Scale-Up Synthesis of Fragrant Nitrogen-Doped Carbon Dots from Bee Pollens for Bioimaging and Catalysis

*Jia Zhang, Yue Yuan, Gaolin Liang, and Shu-Hong Yu**

Supporting Information

1. Experimental Section

Bee pollens were purchased from an online seller on the Taobao website. All chemicals were of analytic grade purity obtained from Sinopharm Chemical Reagent Co. Ltd., and used as received without further purification. Deionized water was used throughout the experiments.

Hydrothermal Synthesis of Carbon Dots from Bee Pollens. The bee pollens were used directly. In a typical experiment, 1 g of bee pollen was dispersed in 40 mL of water under sonication for 5 min. After that, it was transferred into a 50 mL Teflon-lined stainless steel autoclave, sealed, maintained at 180 ^{o}C for 24 h, and then naturally cooled to room temperature. The suspension of carbon dots was obtained after separating the indispersible matters by natural sedimentation and then vacuum filtration (pore size 0.22 μm). Through tuning the amount of feeding bee pollen and the volume of water in a constant ratio, the synthesis can be simply scaled up or down. In the end, the suspension of carbon dots was stored at 4 ^{o}C for further characterization and applications.

Cell Culture and Cytotoxicity Test. A549 human lung adenocarcinoma epithelial cell line and HepG2 human hepatocellular liver carcinoma cell line were obtained from the Molecular Biology Laboratory of Anhui Medical University. Cells were routinely cultured in flasks containing DMEM (High Glucose) supplemented with 10% fetal bovine serum, 100 U mL^{-1} of penicillin, and 100 U mL^{-1} of streptomycin at 37 ^{o}C in a humified hood filled with 5% of CO_2. When cells reached ~85% confluence, they were lifted with trypsin-EDTA, and then averaged among 18 wells in a 24-well plate. 2 mL of medium was added to each well. After overnight culture, solutions of the carbon dots (after passing through 0.22 μm sterile filter membrane) were added to the wells with concentrations at 0, 0.05, 0.1, 0.25, 0.5, and 1 mg/mL, respectively, each of which was repeated in triplicate. The cells were incubated at 37 ^{o}C for 24 h. Afterwards, the cells were again lifted with trypsin-EDTA. Then the supernatant was removed, the cells were resuspended in culture medium and cell number was counted by using a hemocytometer. The mean cell number with error bar with respect to each concentration of carbon dots is an indication of cell proliferation, which is among the several ways to demonstrate the cytotoxicity.

***In Vitro* Fluorescent Cell Imaging.** LoVo human colon carcinoma cells were plated on a 35-mm Petri dish in culture medium one day in advance for cell imaging. For *in vitro* study, cells were incubated in medium containing 0.5 mg/mL of carbon dots for 12 h at 37 ^{o}C, then treated with DMEM for two times and phosphate buffer saline (PBS, pH = 7.4) for one time to wash the unabsorbed free carbon dots. After that, the cells were fixed with paraformaldehyde for at least 10 min, and then imaged on an inverted fluorescence microscope by the use of fluorescence microscope immersion oil, capture under UV, blue, and green excitation.

Catalytic Reduction of Metal Ions to Nanocrystals by Carbon Dots. The reduction of chloroaurate ion by carbon dots was conducted in the presence of sodium citrate at room temperature in dark. Typically, 0.1 mL of carbon dots (7.5 mg/mL) was added to 1 mL of water, followed by the each addition of 25 μL of solution of sodium citrate (50 mg/mL) and hydrogen chloroaurate (50 mM). The catalytic reaction lasted for 1 h before the absorption spectrum taken and other treatment. On the other hand, the reduction of silver ion by carbon dots was conducted at room temperature under direct sunshine irradiation. Typically, 0.2 mL of carbon dots (7.5 mg/mL) was added to 2 mL of water, followed by the addition of 100 μL of solution of silver nitrate (0.1 M), and the reaction lasted for about 5 min under sunshine. Reactions in the absence of carbon dots were taken as controls.

Instruments. UV-vis optical absorption spectra were recorded on a Lambda 25 UV-vis spectrophotometer (PerkinElmer). Photographs were taken with a digital camera. The fluorescence spectra were recorded on a Hitachi F-4600 fluorescence spectrophotometer. Transmission electron microscopy (TEM) and high-resolution TEM images were obtained on a JEOL-2100F transmission electron microscope operating at 200 kV. Time-resolved fluorescence lifetimes were measured by time-correlated single photo counting (TCSPC) on a Horiba JY Fluorolog-3-Tou spectrofluorometer with 370 and 440 nm as the excitation and emission wavelength, individually. Fourier transform infrared (FTIR) spectrum was measured on a Bruker Vector-22 FTIR spectrometer from 4000 to 400 cm^{-1} at room temperature. X-ray photoelectron spectroscopy (XPS) study was performed on an ESCALAB 250 spectrometer (VG Scientific). Peak positions were internally referenced to the C1s peak at 284.6 eV. X-ray powder diffraction (XRD) patterns were obtained on a Philips X'Pert PRO SUPER X-ray diffractometer equipped with graphite-monochromatized Cu Kα radiation. The Zeta potential measurement was

performed on a Malvern Zetasizer instrument (Malvern, UK). The elemental contents of C, H, O, and N in bee pollen and carbon dot were measured on a Vario EL III elemental analyzer system (Germany). Atomic force microscopy (AFM) measurements were carried out with an AJ-III (Shanghai Aijian Nanotechnology, China) using a tapping mode. Both bright-field image and fluorescent microscope images were taken on an inverted fluorescent microscope (OLYMPUS IX71).

2. Supporting Figures

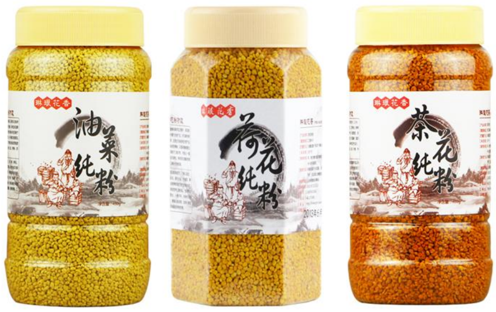

Figure S1. From left to right are the photos of each bottle of rapeseed flower bee pollen, lotus bee pollen, and camellia bee pollen, respectively.

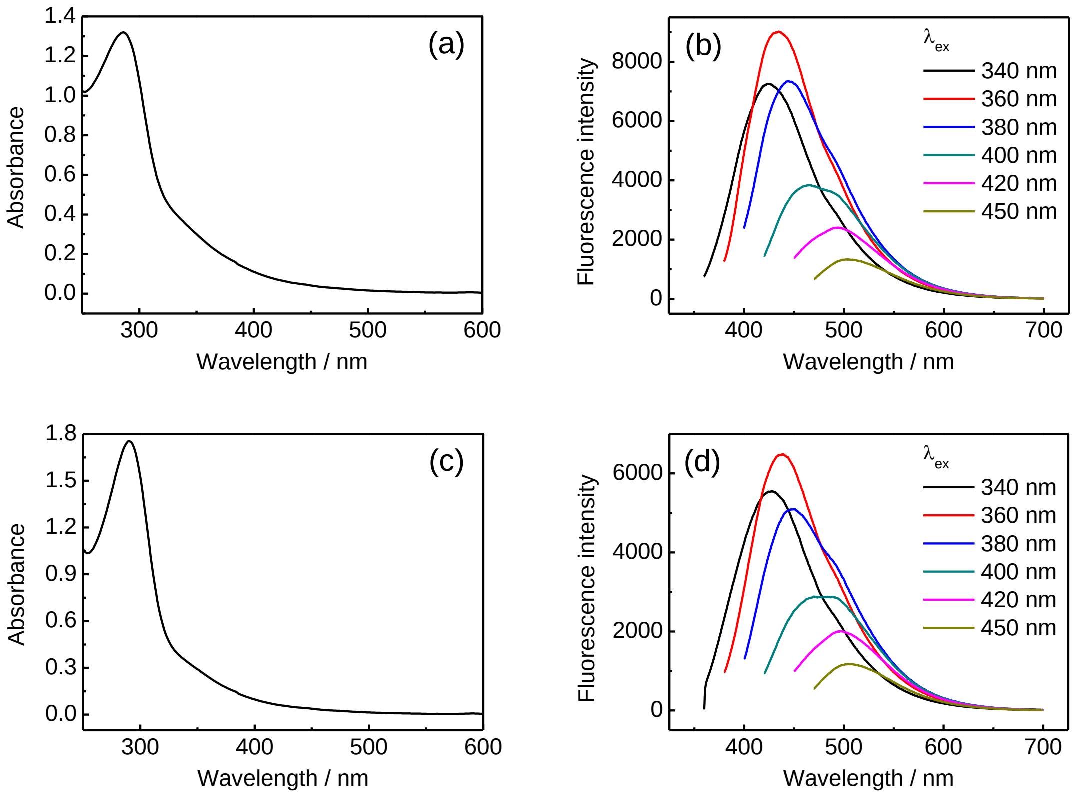

Figure S2. The absorption spectra (a, c) and wavelength-dependent emission spectra (b, d) of c-CDs (a, b) and l-CDs (c, d).

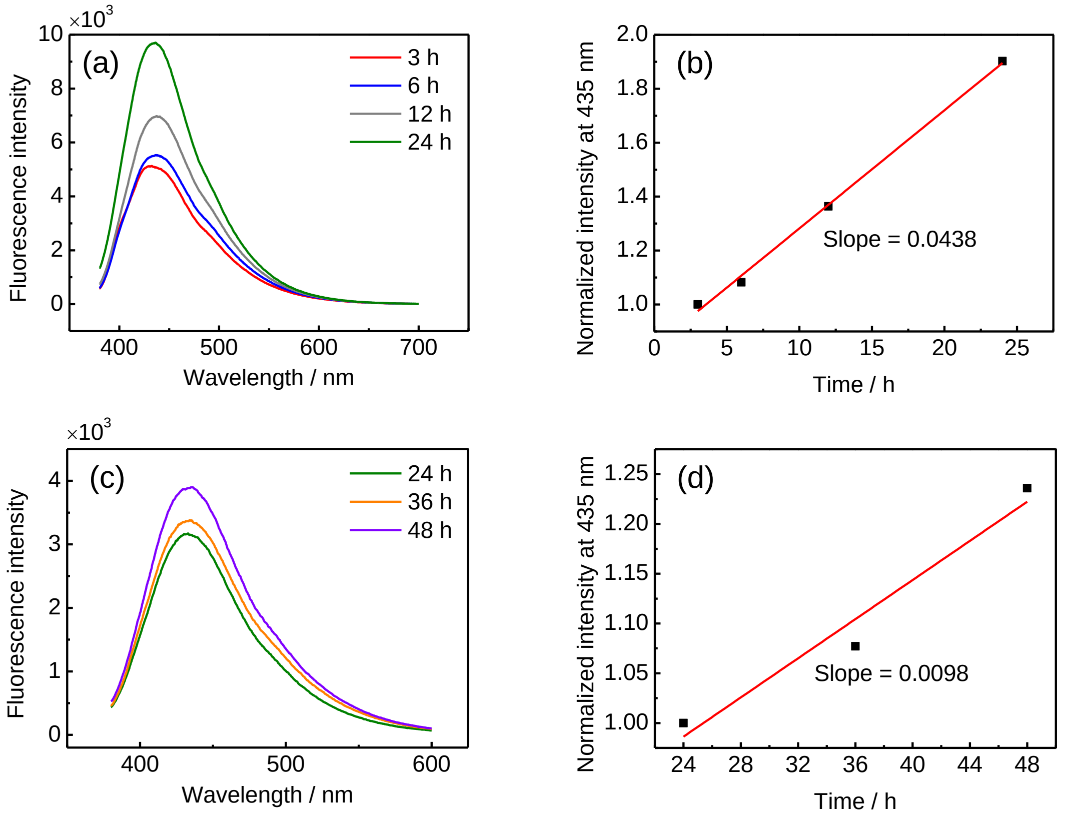

Figure S3. (a, c) Fluorescent emission spectra of r-CDs for different reaction time upon excitation at 360 nm and (b, d) the correlation of fluorescence intensity *versus* time.

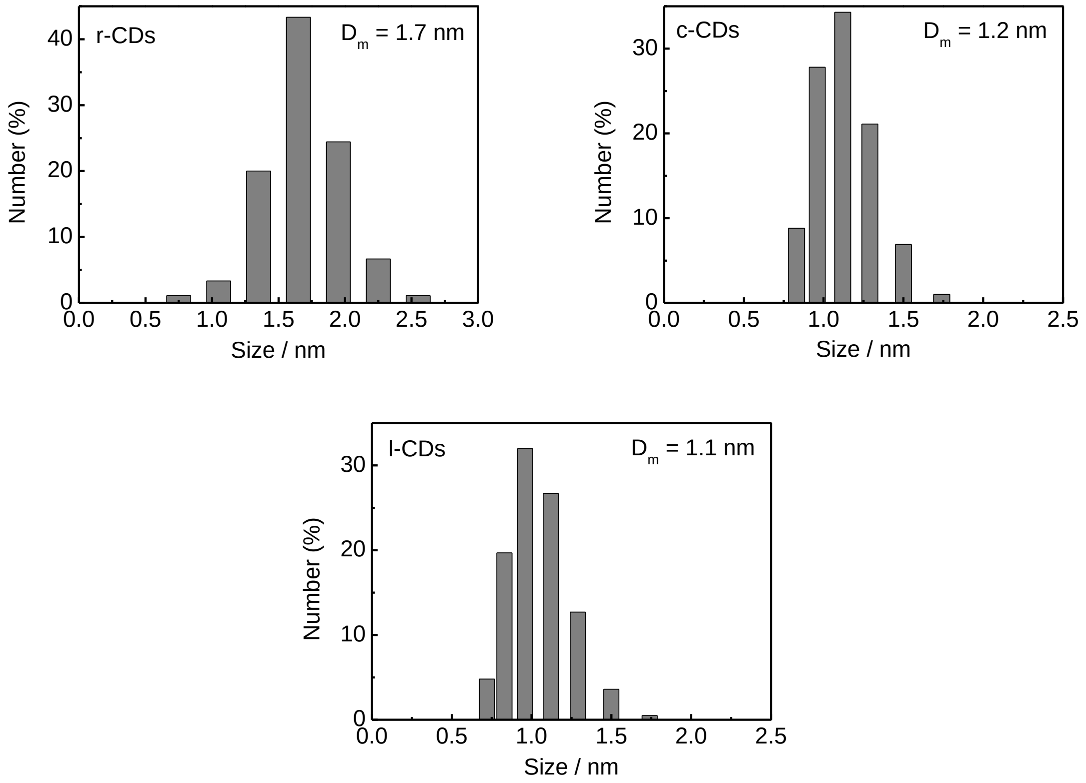

Figure S4. Size distribution of carbon dots derived from the three bee pollens.

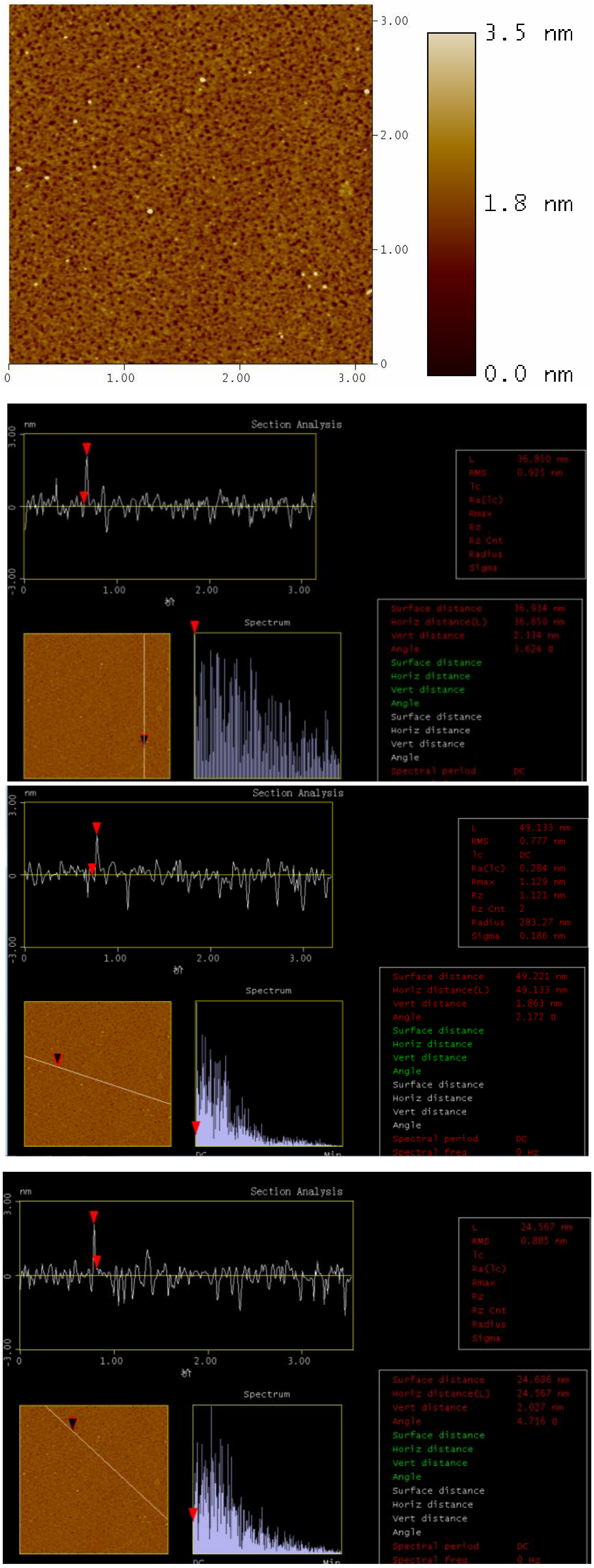

Figure S5. An AFM image of r-CDs and the height evaluation.

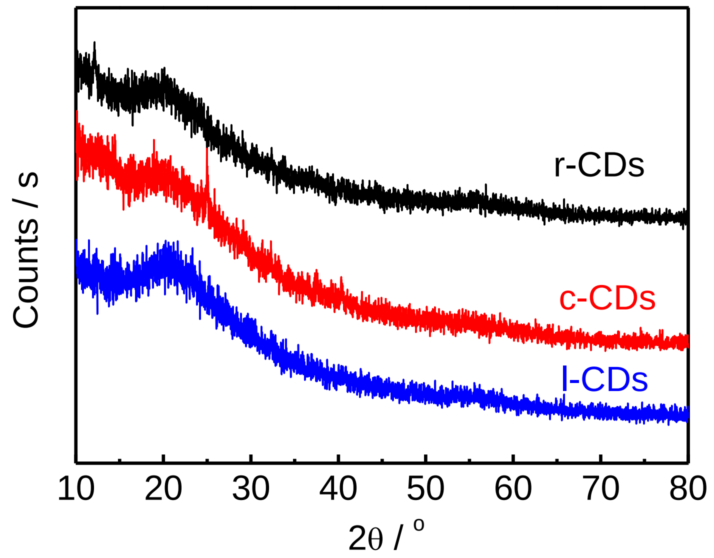

Figure S6. XRD patterns of carbon dots derived from the three bee pollens.

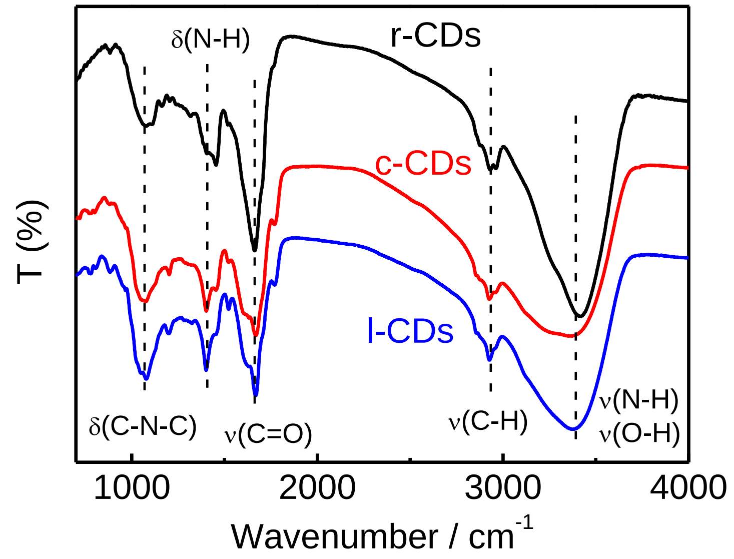

Figure S7. FTIR spectrum of carbon dots derived from the three bee pollens.

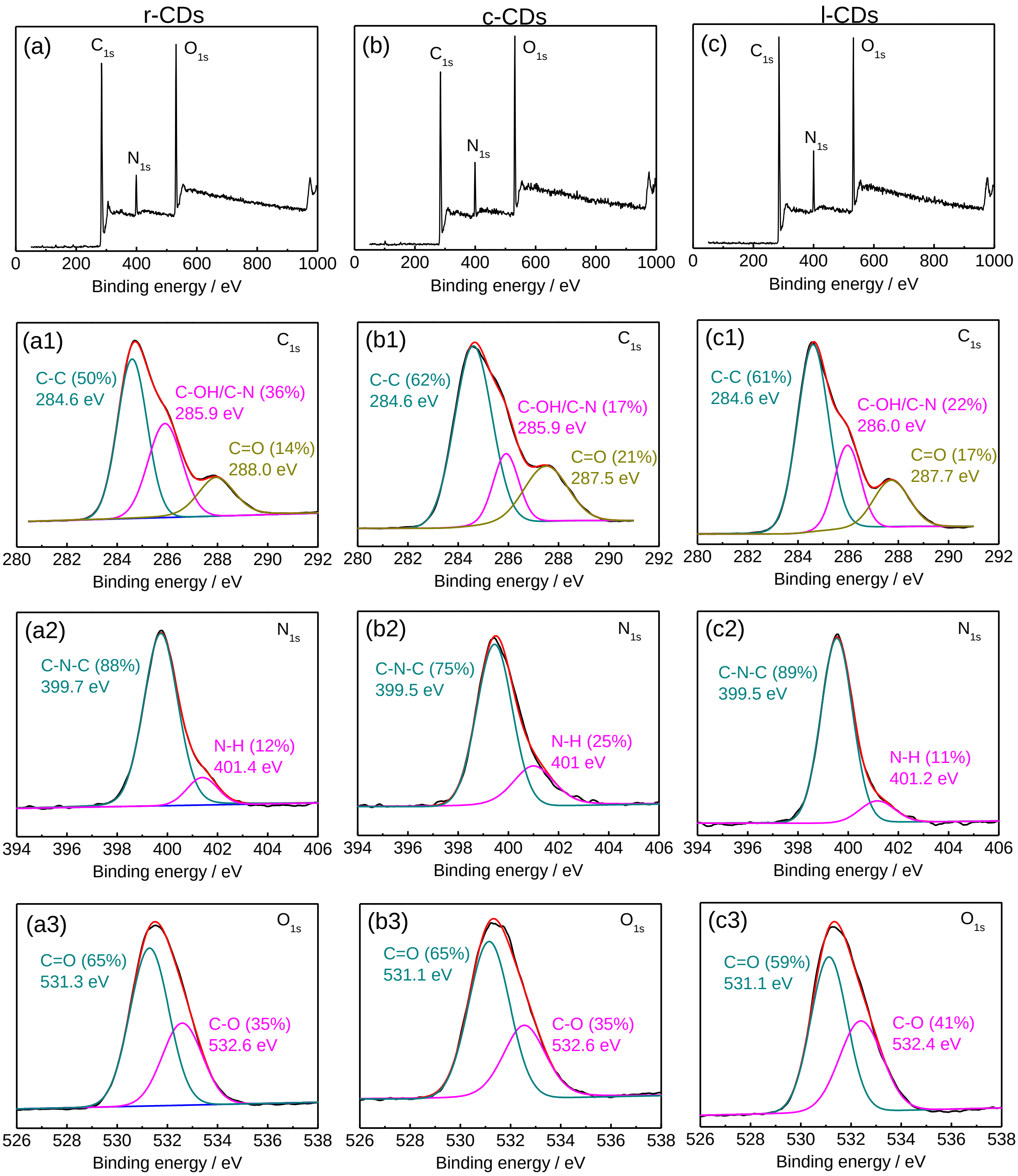

Figure S8. XPS survey spectra for the three carbon dots and the deconvoluted spectra for C_{1s}, N_{1s}, and O_{1s} binding energy.

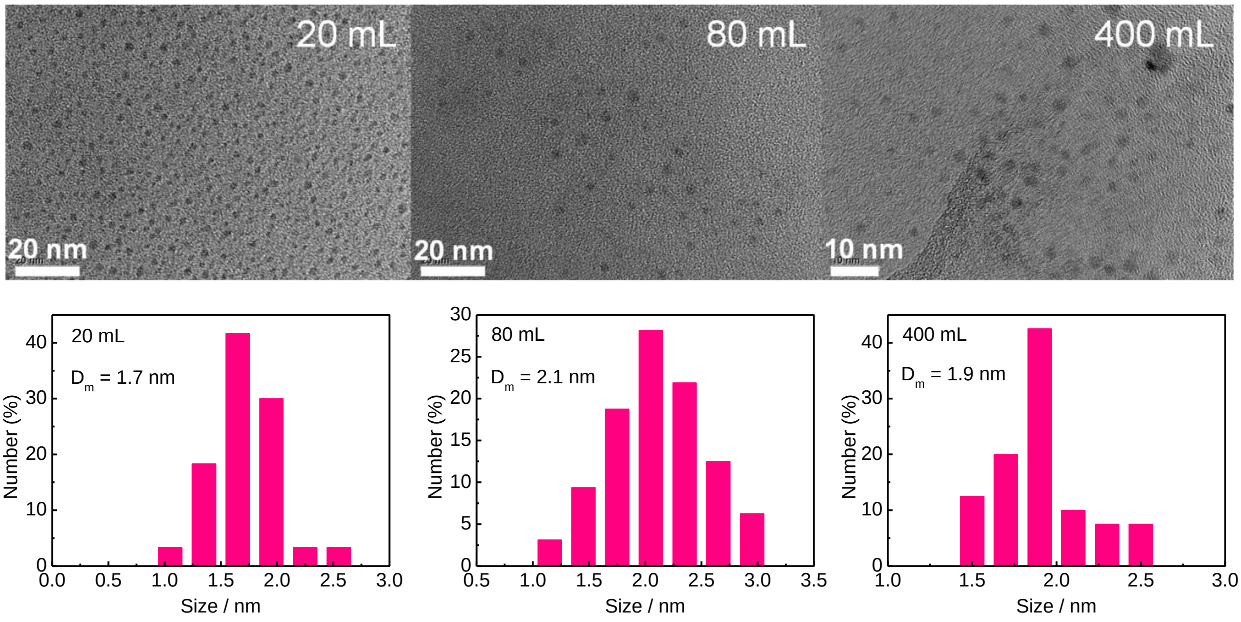

Figure S9. TEM images of r-CDs prepared at specific volumes and their corresponding size distribution.

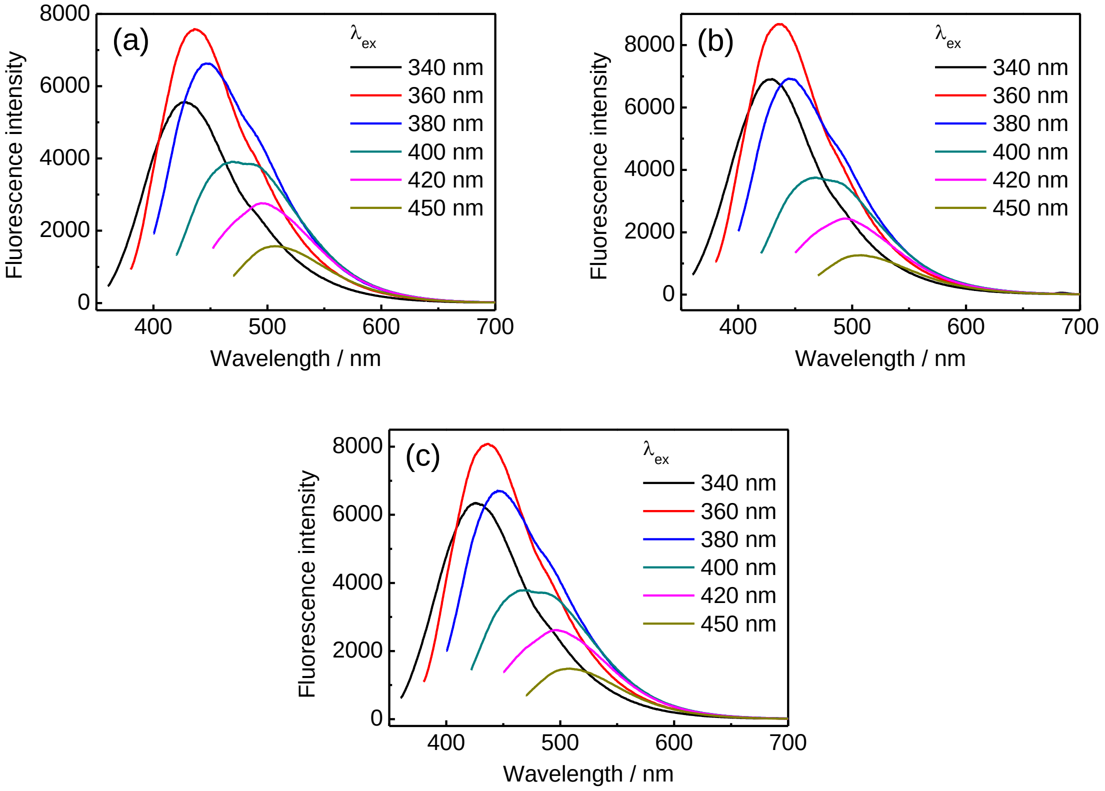

Figure S10. Wavelength-dependent fluorescent emission spectra for r-CDs prepared at specific volumes: (a) 20 mL, (b) 80 mL, and (c) 400 mL.

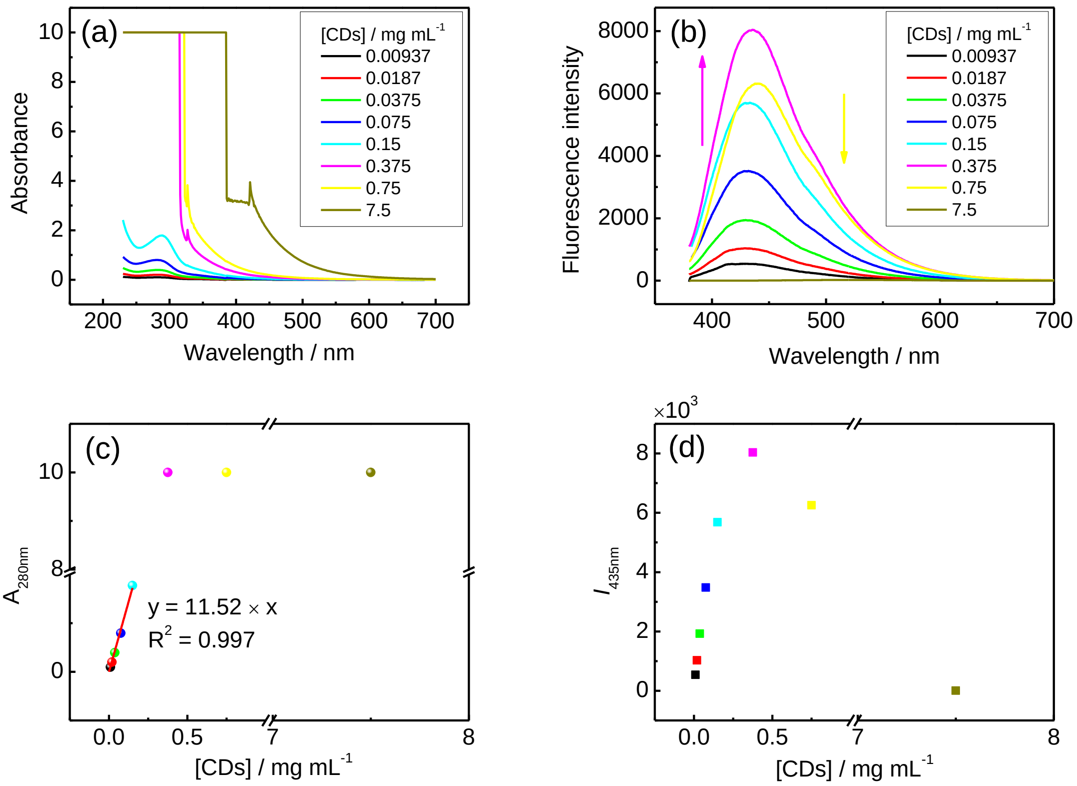

Figure S11. (a) Absorption spectra and (b) fluorescent emission spectra for r-CDs at different concentrations. Correlation of (c) absorbance at 280 nm and (d) fluorescence intensity at 435 nm with the concentration of carbon dots.

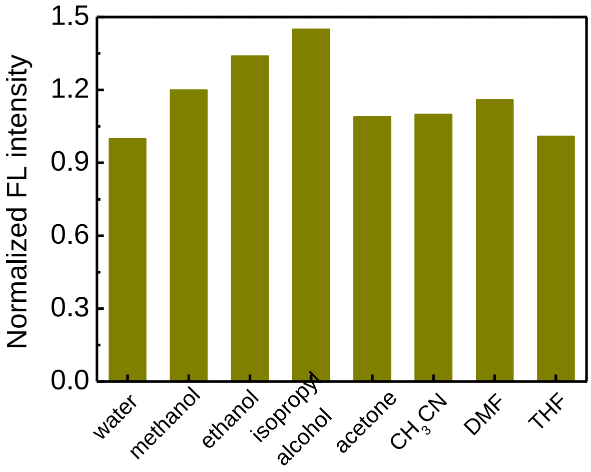

Figure S12. Fluorescence intensity of r-CDs in other solvent compared with that in water.

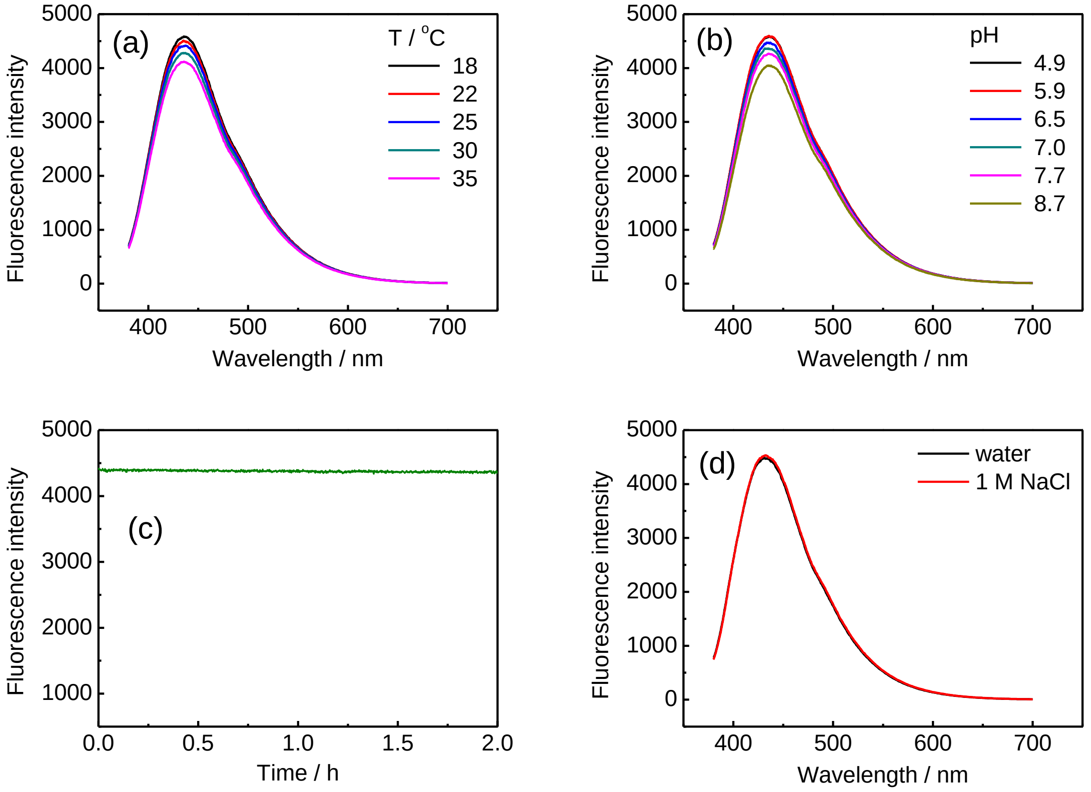

Figure S13. Fluorescent emission spectra of suspension of r-CDs at different (a) temperature and (b) solution pH. (c) Photostability test of r-CDs under continuous excitation for 2 h. (d) Stability test of r-CDs in the presence of highly concentrated salt.

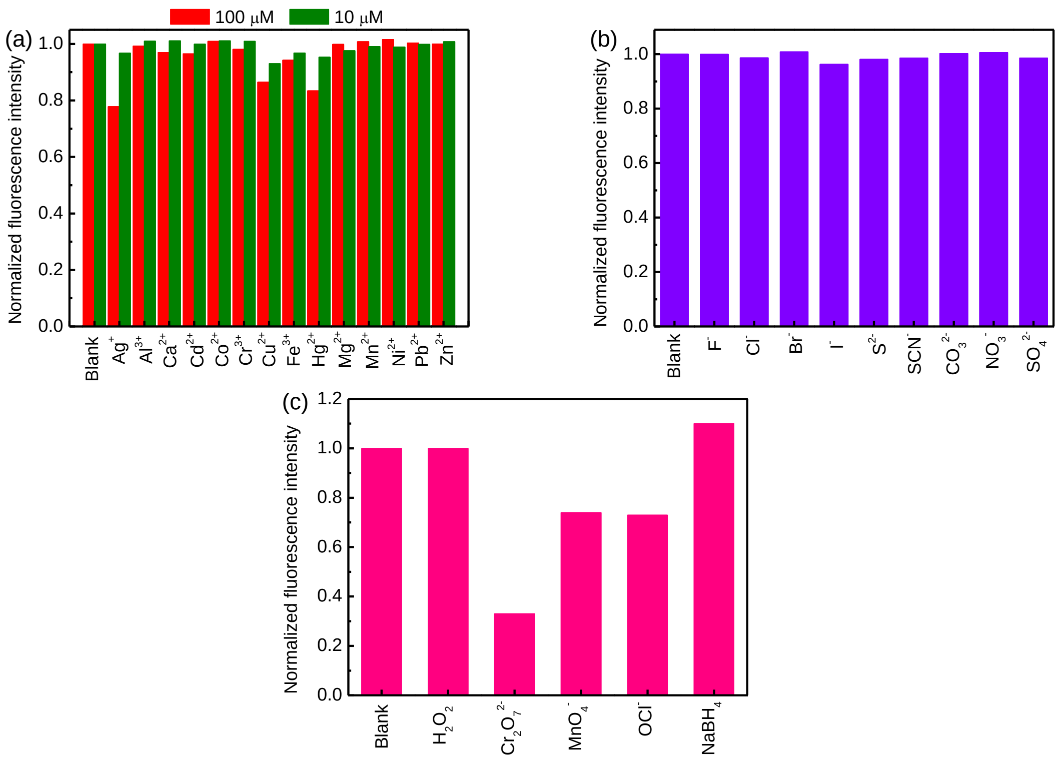

Figure S14. Influence of (a) metal ions, (b) anions, and (c) other species on the fluorescence intensity of suspension of carbon dots. For (b, c), the concentrations of species were all 100 μM.

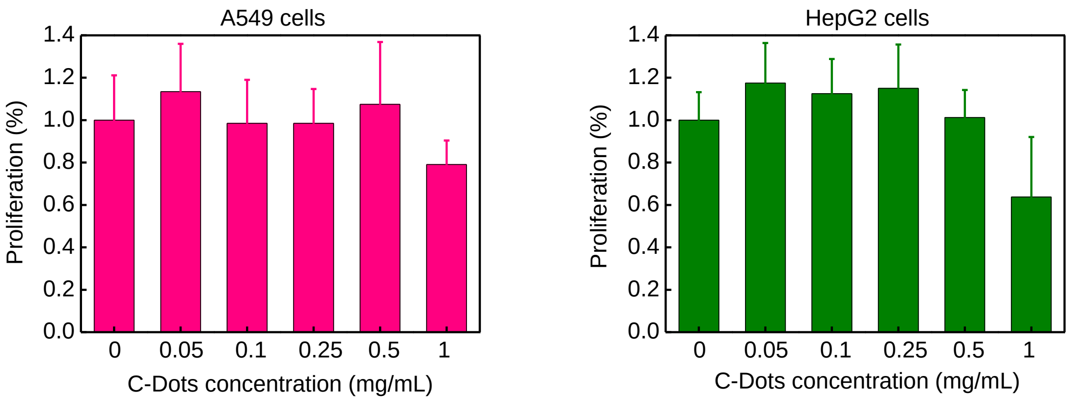

Figure S15. Cytotoxicity test of r-CDs by cell proliferation with A549 human lung adenocarcinoma epithelial cell line and HepG2 human hepatocellular liver carcinoma cell line.

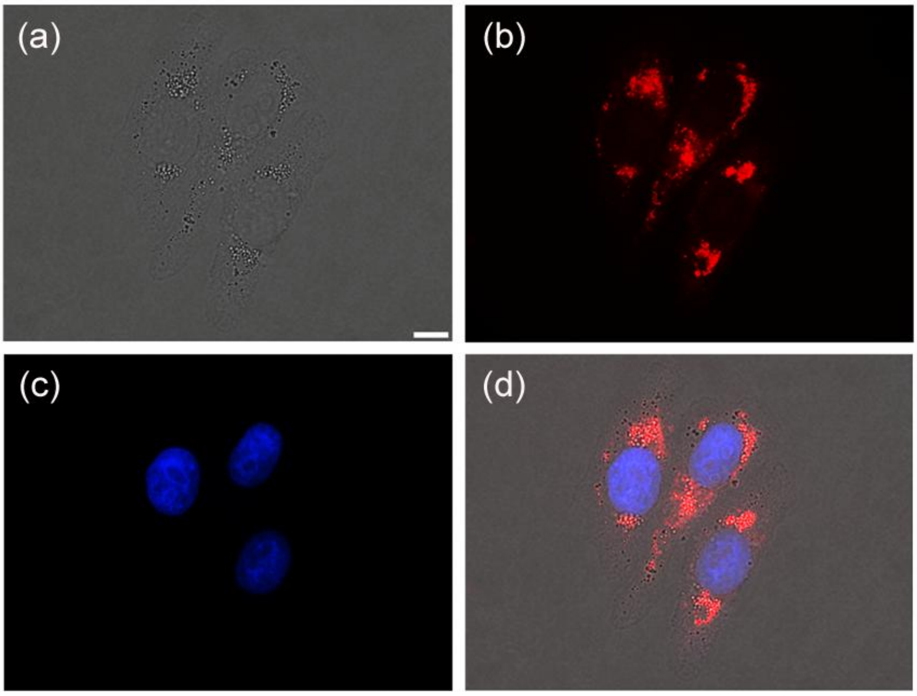

Figure S16. Bright-field image (a) and fluorescent images (b, c) of LoVo cell after incubation with r-CDs (0.5 mg/mL) for 12 h at 37 °C and then fixed by paraformaldehyde to be stained with DAPI. Fluorescent image (b) was captured under green excitation, and fluorescent image (c) of cell nuclei stained with DAPI was captured under UV excitation. (d) merged image of (b) and (c). Scale bar: 10 μm.

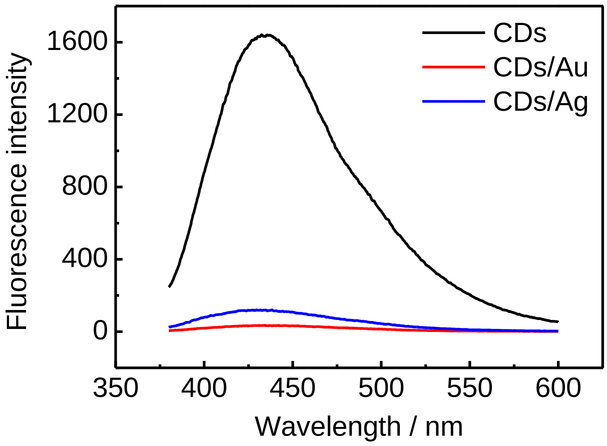

Figure S17. Fluorescence changes of r-CDs solution after reaction with metal ions.

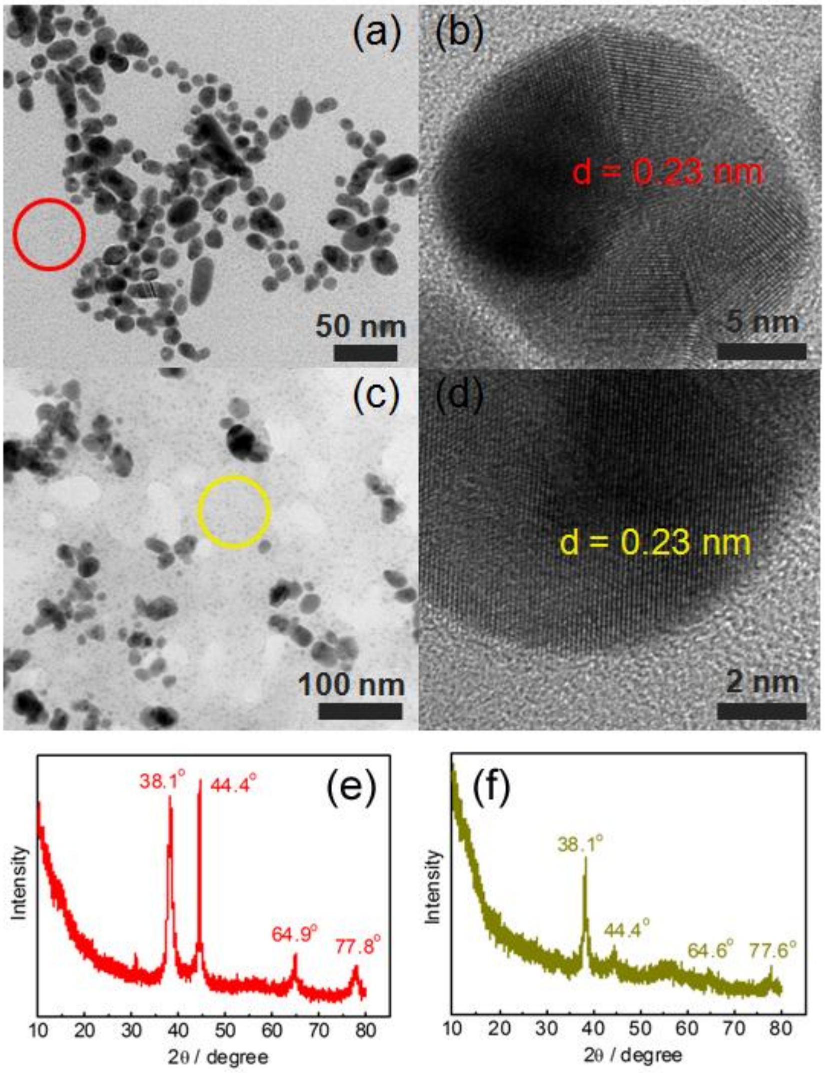

Figure S18. TEM and HRTEM images for the CDs/Au (a, b) and CDs/Ag (c, d). Within the circles marks the presence of carbon dots. Powder XRD patterns of the CDs/Au (e) and CDs/Ag (f) after centrifugation and drying.

3. Supporting Tables

Table S1. A collection of biomass as carbon precursors to prepare carbon dots with differed quantum yield (QY), particle size, and production yield.

Biomass	Coffee grounds	*Bombyx mori*silk	Cocoon silk	Banana juice	Orange juice	Soy milk	Egg	Plant leaf	Pomelo peel	Bee pollens
QY / %	3.8	13.9	38	8.95	26	2.6	6(white) 8(yolk)	16.4	6.9	6.1 ~ 12.8
Size / nm	5	5	70	3	2.5	25	3.4(white) 2.2(yolk)	3.7	3	1.1 ~ 2.1
Yield / %	12 ±3	?	?	58	?	?	5.96	?	?	28 ~ 38
Reference	S1	S2	S3	S4	S5	S6	S7	S8	S9	This work

Note: The question mark signifies that no data were provided by the references.

Table S2. Elemental analysis of rapeseed flower bee pollen and the three carbon dots (24 h).

Element	C / %	H / %	N / %	O / %	QY / %
r-Bee pollen	42.97	7.39	3.89	39.35	Low
r-CDs	52.30	6.67	7.10	32.52	9.1
c-CDs	44.69	6.44	6.67	40.82	8.9
l-CDs	41.30	5.91	4.28	46.70	6.1

Table S3. Fluorescent lifetime of the three carbon dots derived from bee pollens. Note that

$$\tau_{average} = \frac{\sum^{2}_{i=1} A_i \tau_i^2}{\sum^{2}_{i=1} A_i \tau_i}$$

Lifetime	τ_1 (ns)	A_1	τ_1 (ns)	A_2	$\tau_{average}$ (ns)
r-CDs	3.29	0.84	12.7	0.16	7.28
c-CDs	3.14	0.80	12.1	0.20	7.54
l-CDs	3.51	0.80	12.3	0.20	7.61

4. Supporting References

S1. P. C. Hsu, Z. Y. Shih, C. H. Lee, H. T. Chang, *Green Chem.* **2012**, *14*, 917.

S2. Z. L. Wu, P. Zhang, M. X. Gao, C. F. Liu, W. Wang, F. Leng, C. Z. Huang, *J. Mater. Chem. B* **2013**, *1*, 2868.

S3. W. Li, Z. H. Zhang, B. A. Kong, S. S. Feng, J. X. Wang, L. Z. Wang, J. P. Yang, F. Zhang, P. Y. Wu, D. Y. Zhao, *Angew. Chem. Int. Ed.* **2013**, *52*, 8151.

S4. B. De, N. Karak, *RSC Adv.* **2013**, *3*, 8286.

S5. S. Sahu, B. Behera, T. K. Maiti, S. Mohapatra, *Chem. Commun.* **2012**, *48,* 8835.

S6. C. Z. Zhu, J. F. Zhai, S. J. Dong, *Chem. Commun.* **2012**, *48*, 9367.

S7. J. Wang, C. F. Wang, S. Chen, *Angew. Chem. Int. Ed.* **2012**, *51*, 9297.

S8. L. Zhu, Y. Yin, C.-F. Wang, S. Chen, *J. Mater. Chem. C* **2013**, *1*, 4925.

S9. W. B. Lu, X. Y. Qin, S. Liu, G. H. Chang, Y. W. Zhang, Y. L. Luo, A. M. Asiri, A. O. Al-Youbi, X. P. Sun, *Anal. Chem.* **2012**, *84*, 5351.
